# Dynamic Magnetostatic Energy Correction Based on Domain Area Evolution for Mesoscopic Hysteresis Modeling

**DOI:** 10.3390/ma19122659

**Published:** 2026-06-20

**Authors:** Mengxing Li, Yao Ying, Jing Yu, Jingwu Zheng, Juan Li, Liang Qiao, Akihisa Inoue, Shenglei Che

**Affiliations:** 1 College of Materials Science and Engineering, Zhejiang University of Technology, Hangzhou 310014, China; lmx_lmx2021@163.com (M.L.); yying@zjut.edu.cn (Y.Y.); yujing@zjut.edu.cn (J.Y.); zhengjw@zjut.edu.cn (J.Z.); juanli@zjut.edu.cn (J.L.); lqiao@zjut.edu.cn (L.Q.); 2 Research Center of Magnetic and Electronic Materials, Zhejiang University of Technology, Hangzhou 310014, China; inoue@jiu.ac.jp; 3 International Institute of Green Materials, Josai International University, Togane 283-8555, Japan

**Keywords:** hysteresis loop, domain energy, domain area, magnetostatic energy, demagnetizing field, mesoscopic model, electrical steel sheet

## Abstract

In mesoscopic domain energy models of electrical steel sheets, the demagnetizing field ***H***_d_ is usually held constant at its domain-wall-complete value throughout magnetization. This treatment overestimates the magnetostatic energy at intermediate states and distorts the simulated hysteresis loop. We introduce a field-dependent coefficient υ_H_ that scales the magnetostatic energy at each field step. The coefficient is calculated from the aligned-domain area measured by magneto-optical Kerr microscopy and is anchored at the negative coercivity point *H* = −*H*_c_, where the macroscopic magnetization vanishes and the aligned-domain area S_0_ is minimal. The definition follows from the linear relation *H*_d_ ≈ ***N***_d_·***M*** that holds during domain-wall motion. Measurements in two observation zones of a grain-oriented steel give consistent υ_H_ curves, confirming the repeatability of the method. When the correction is incorporated into an Assembly Domain Structure Model, the coercivity error drops from 113% to 9–22% relative to the experimental average, with the predicted value falling inside the experimental range, and the remanence error drops from 39.9% to 15–17%. The same correction, applied to a second grain-oriented steel of a different grade, likewise reduces the coercivity and remanence errors (to about 23% and 18%, respectively), confirming that the method is applicable across grades.

## 1. Introduction

The hysteresis behavior of ferromagnetic materials is a fundamental property, and modeling it accurately is essential for the design of power equipment [1,2]. Among soft-magnetic materials, grain-oriented and non-oriented electrical steel sheets remain the dominant choice for transformer cores and rotating machines, thanks to their low core loss, high permeability, and good manufacturability [3]. Current modeling approaches fall into two main groups. Macroscopic phenomenological models, such as the Preisach model, can achieve good accuracy but at the cost of extensive parameter identification from large families of measured loops [4]; recent variants such as scalar Preisach models with descending-branch identification improve efficiency yet still rely on substantial experimental input [5]. In contrast, mesoscopic physical models based on domain energy, such as the Assembly Domain Structure Model (ADSM) [6,7], ground the calculation in domain-scale physics and have therefore attracted growing attention. These models obtain the magnetization state by minimizing the total domain energy, drawing on energy-based frameworks that connect domain-scale physics to the macroscopic magnetic response [8,9]. The total energy typically includes contributions from the Zeeman interaction, magneto-crystalline anisotropy, magnetostatic interaction, and hysteresis dissipation [10,11].

A long-standing issue in these mesoscopic models is how they handle the magnetostatic energy. In most implementations, the demagnetizing field ***H***_d_ is treated as a constant equal to its value at the domain-wall-complete state, and this constant is used throughout the entire hysteresis loop calculation. In reality, however, ***H***_d_ is not constant. As domain walls sweep through the material and the aligned-domain fraction grows, the net surface pole density rises, and so does ***H***_d_, until domain-wall motion is complete [12]. This progressive change in the demagnetizing field has been noted in energy-based hysteresis frameworks that distinguish between intermediate and saturated magnetization states. Fixing ***H***_d_ at its maximum value for all intermediate states therefore overestimates the magnetostatic energy and places an artificially large demagnetizing pressure on the growing aligned domains. The result is an overestimated coercivity and an underestimated remanence—both critical parameters for electromagnetic device design.

The evolution of magnetostatic energy during magnetization is thus tied to domain-wall motion. If this coupling can be captured from experimental domain observations, the magnetostatic energy can be corrected at each field step without performing a full grain-by-grain demagnetization-field calculation, which the original ADSM requires [7]. Following this idea, the present work corrects the magnetostatic energy using the domain area measured during magnetization. A magneto-optical Kerr microscope is used to record the magnetization process in two single-grain regions of a grain-oriented electrical steel sheet, and the aligned-domain area is extracted as a function of the applied field. From these data a field-dependent correction coefficient υ_H_ is derived and inserted into the mesoscopic model to scale the magnetostatic energy. Simulation results of the corrected model are then compared with measured hysteresis loops, and the consistency of υ_H_ between the two regions demonstrates the repeatability of the method. The same procedure is then applied to a second grain-oriented steel of a different grade to verify that the correction is not restricted to a single material.

## 2. Domain Observation and Domain Area Dynamic Feature Extraction

### 2.1. Domain Observation

**Figure 1:**
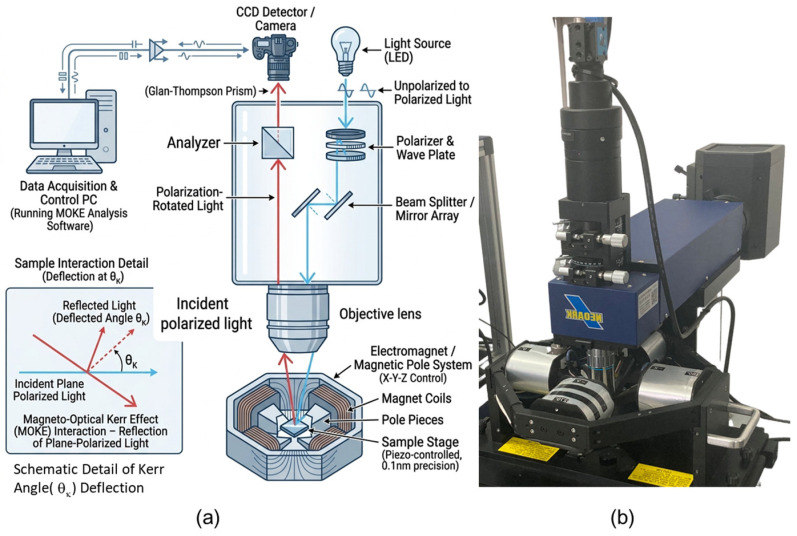
Magneto-Optical Kerr microscope: (**a**) Schematic diagram; (**b**) Physical map.

To capture the dynamic domain evolution under an applied field and extract the necessary mesoscopic parameters, a BH782PI-SHG magneto-optical Kerr microscope (Neoark Corporation, Hachioji, Japan) was employed (Figure 1). Relying on the rotation of polarized light reflected from a magnetized surface, the Kerr effect provides a reliable, non-destructive approach for observing domain dynamics in electrical steels [13,14]. The experimental apparatus pairs an electromagnet with a polarized optical system. Two commercial grain-oriented silicon steels of different grades were used in this study, denoted Sample A (a high-permeability grain-oriented steel, ~0.23 mm thick) and Sample B (a conventional grain-oriented steel, ~0.30 mm thick), both being Fe–Si alloys containing approximately 3 wt% Si with the Goss texture {110} <001>. Prior to observation, each sample was cut to 6 mm × 6 mm and polished, then mounted at the center of the magnetic field. Sample A served as the primary sample for domain observation and model development, while Sample B was used for the cross-sample applicability verification in Section 4.3.2.

The magnetic field was applied along the rolling direction of the sample, generated by the four-pole electromagnet system of the Kerr microscope; it was applied quasi-statically in steps of 5 A/m, with the sweep covering the complete magnetization-reversal process from the positive single-domain saturation state to the negative single-domain saturation state (the maximum field amplitude differing between the two samples). The rolling direction was chosen for the following reasons.

First, the model is ultimately validated against the macroscopic hysteresis loop measured along the rolling direction; applying the field along the same direction in the Kerr observation ensures that the extracted domain area and the macroscopic loop used for comparison correspond to the same magnetization direction and are therefore comparable.

Second, the <001> easy axis of grain-oriented silicon steel is strongly aligned with the rolling direction, so that field application along it yields a magnetization reversal dominated by easy-axis domain-wall motion, with a clearly resolved evolution of the domain structure that facilitates quantitative extraction of the domain area; deviating from the rolling direction would introduce more complex processes such as domain rotation.

Third, the present model represents the entire sample by a single representative grain, which requires the grains to respond to the field in essentially the same way; the Goss texture aligns one <001> easy axis of every grain closely with the rolling direction, so that under field application along this direction the orientation of the field relative to the easy axis is, to a good approximation, the same for all grains and their responses are closely similar, allowing description by a single representative grain. If the field deviates from the rolling direction, the orientation differences in the grains about the rolling direction make their responses markedly non-uniform, and a single representative grain no longer suffices.

**Figure 2:**
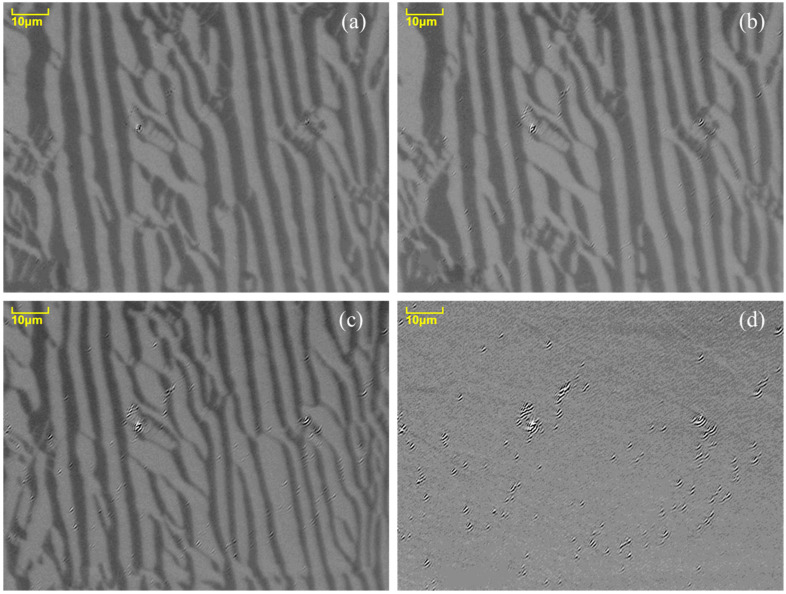
Magnetic domain image of zone 1 (Sample A): (**a**) Initial multi-domain state; (**b**) Domain wall motion; (**c**) Major domain expansion; (**d**) Single-domain saturation.

**Figure 3:**
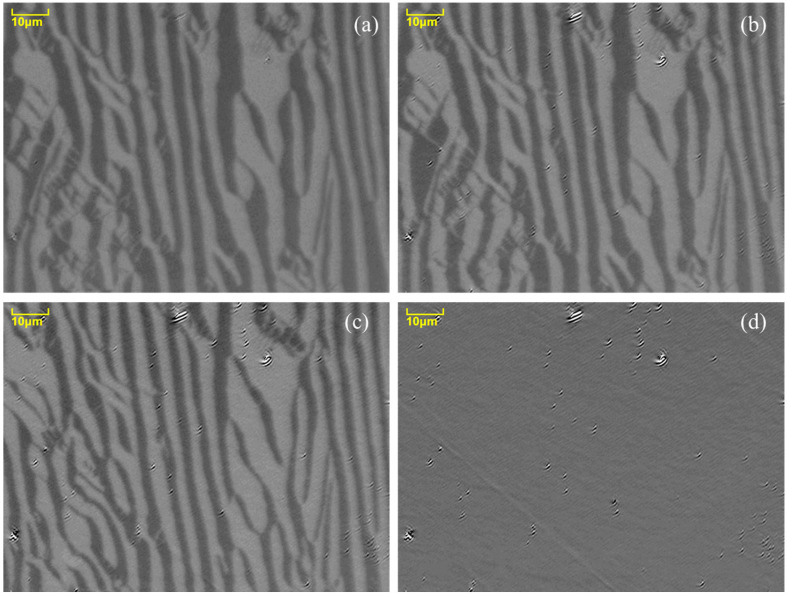
Magnetic domain image of zone 2 (Sample A): (**a**) Initial multi-domain state; (**b**) Domain wall motion; (**c**) Major domain expansion; (**d**) Single-domain saturation.

Figure 2 and Figure 3 show the magnetization process at the mesoscopic scale recorded by the Kerr microscope in two different zones of the sample A, with the magnetic field applied along the rolling direction. Bright and dark stripe patterns represent domains whose magnetic moments point in directions close to and far from the external field direction, respectively. As the field increases from (a) to (d), the bright-domain area grows progressively until a single bright domain fills the field of view, indicating that domain-wall movement is complete.

### 2.2. Extraction of Domain Area Information

**Figure 4:**
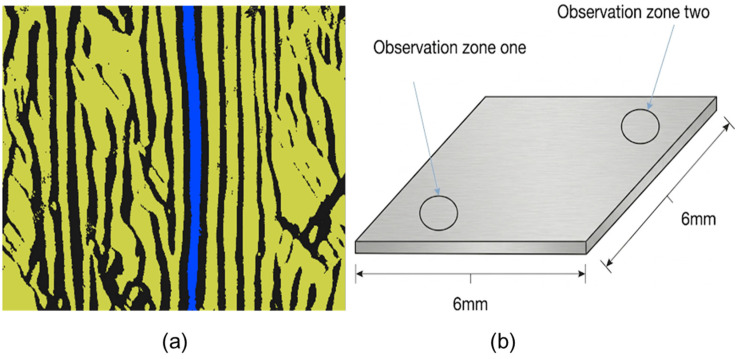
Extraction of magnetic domain area information: (**a**) Extraction schematic; (**b**) Extracted region.

To quantify the mesoscopic magnetization process, the domain area aligned with the external field direction was extracted from the Kerr images using the built-in domain-area acquisition function of the BH782PI-SHG Kerr microscope (Neoark Corporation, Hachioji, Japan). Rather than relying on manual tracing, the extraction is carried out automatically by this standardized instrument routine, with the following steps: (i) the image is differenced against the domain-wall-completion reference image to remove residual scratches and imaging artifacts; (ii) the contrast of the differential image is enhanced by the routine’s fixed default settings (a +20% gain for darker regions and a −80% gain for brighter regions, applied automatically rather than set by the operator); (iii) a target domain type is specified, after which the connected regions belonging to the selected target domain (blue areas) and the other domains of the same orientation type across the image (yellow areas) are identified automatically. The studied domain area is the sum of these two contributions (Figure 4a). Because the procedure relies on a standardized, instrument-level function with default parameters rather than operator-dependent segmentation, it can be reproduced by any group using the same or an equivalent domain-area acquisition tool.

**Figure 5:**
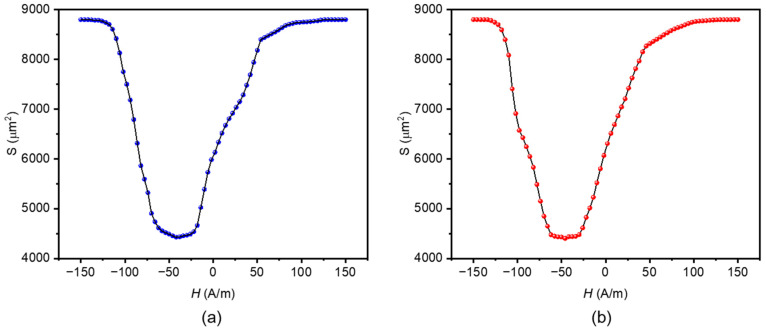
Domain area changes in the two zones (Sample A): (**a**) Zone 1; (**b**) Zone 2.

The two observation zones (Figure 4b) were not selected arbitrarily [15]: each lies within a single grain with a clearly resolved domain structure and minimal surface artifacts, ensuring reliable area extraction; moreover, the two zones were deliberately taken from different grains, and the consistency of their domain-area curves (Figure 5) shows that the single-grain measurement is representative of the sample, thereby providing a preliminary verification of the repeatability of the method described below.

Figure 5 shows the domain-area curves obtained from each zone as a function of the applied field *H*. The domain-area curves S*_H_*(*H*) from regions 1 and 2 share a similar overall shape, both exhibiting a characteristic “U-shaped” profile. In the high-field regime (|*H*| > 100 A/m), the domain area saturates, with the saturation value reaching approximately 8800 μm^2^ in both regions. The curves are not symmetric about *H* = 0; their minima occur near H ≈ −40 to −50 A/m rather than at zero field. At *H* = 0 the sample retains a net remanent magnetization *B*_r_, whereas the position of the minimum domain area corresponds to the coercive field *H*_c_, at which the macroscopic magnetization vanishes and the sample is in a demagnetized state. The minimum domain areas in both regions lie in the range 4400–4500 μm^2^, about half of the saturation value, indicating that the areas of the positively and negatively oriented domains are nearly equal—consistent with the demagnetized state at *H*_c_. The coercive-field positions, however, differ slightly between the two regions, being −42 A/m for region 1 and −46 A/m for region 2. This small discrepancy can be attributed to local variations between the two grains—in particular in the local distribution of defects and pinning sites—rather than to a substantial difference in crystallographic orientation, consistent with the highly aligned Goss texture of the grain-oriented steel. In addition, within the field intervals of −100 to −50 A/m and −20 to +50 A/m, the curves display steep slopes corresponding to magnetization reversal driven by rapid domain-wall motion; the slopes on either side are comparable, suggesting that the forward and reverse magnetization processes proceed with similar ease. It is worth noting that regions 1 and 2 were taken from two different grains observed at the single-grain scale, yet their saturation levels, characteristic field positions, and overall curve shapes remain in close agreement, indicating that the magnetization-reversal behavior is highly consistent among the grains examined in this study.

## 3. Correction of Magnetostatic Energy Considering Changes in Domain Area

### 3.1. Physical Basis of the Correction

To reduce the magnetostatic energy arising from the free surface poles produced under a single magnetization direction [12], the material spontaneously subdivides into multiple domains of alternating magnetization; the magnetostatic energy is therefore a necessary condition for domain formation, expressed in simplified form as

(1) $$W_{st}=-\frac{\mu_{0}M_{s}}{2}H_{d}\cdot m\;,$$

where μ_0_ is the vacuum permeability, M_s_ is the saturation magnetization, ***m*** = (*m*_x_, *m*_y_, *m*_z_) is the unit magnetization vector sum of the domain, and ***H***_d_ is the demagnetizing field.

**Figure 6:**
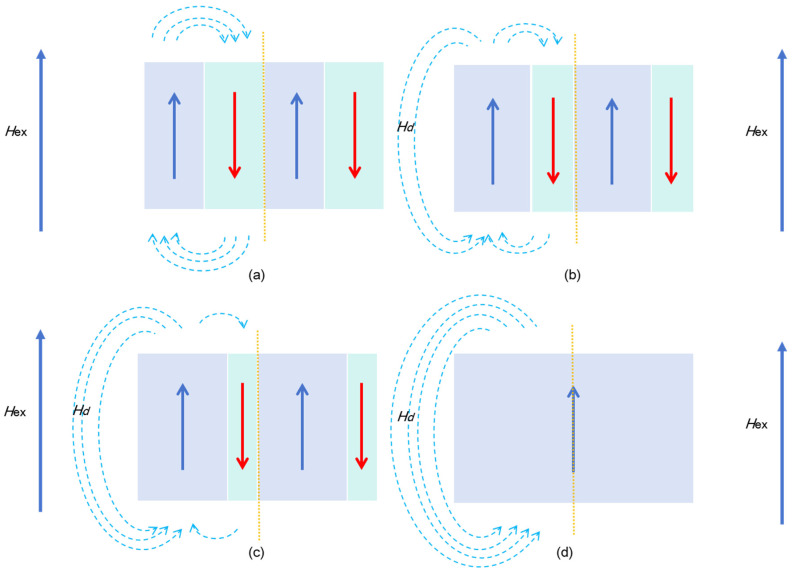
The demagnetizing field changes with the magnetic field during the magnetization process (left-right symmetrical structure): (**a**) Initial multi-domain state; (**b**) Domain wall motion; (**c**) Major Domain expansion; (**d**) Single-domain saturation.

An important point about Equation (1) is that ***H***_d_ is not constant but evolves during magnetization. As the applied field *H* increases, domain-wall motion enlarges the area of domains aligned with *H*, raising the net surface-pole density; by the relation ***H***_d_ ≈ ***N***_d_·***M*** [11], ***H***_d_ grows in proportion to the net magnetization (Figure 6). The magnetostatic energy therefore keeps increasing from the demagnetized state until domain-wall motion is complete, where ***H***_d_ reaches its maximum. Fixing ***H***_d_ at this maximum value over the whole loop, as in the simplified model, overestimates the magnetostatic energy at intermediate fields and distorts the computed hysteresis loop.

### 3.2. Derivation of the Correction Coefficient

The correction connects the measured domain area to the magnetostatic energy at each field step. For a sample of fixed geometry, the demagnetizing field is proportional to the net magnetization, ***H****_d_* ≈ ***N****_d_*·***M***. During domain-wall motion, the net magnetization is in turn approximately proportional to the fraction of aligned-domain area. The growth of the aligned-domain area from the demagnetized reference state to domain-wall completion therefore provides a direct experimental measure of how ***H****_d_* evolves, allowing the magnetostatic energy to be scaled accordingly at each field step.

Two field values serve as references for correction coefficient υ_H_. At the domain-wall-completion field *H* = ±*H_t_*, the sample is essentially single-domain, the magnetic moments are aligned with *H*, and the magnetostatic energy attains its physically meaningful maximum. At the negative coercivity point *H* = −*H_c_*, the macroscopic magnetization vanishes and the aligned-domain area reaches its minimum *S_0_*. The coefficient υ_H_ measures the fraction of domain-wall motion completed between these two states.

The correction coefficient υ_H_ is defined piecewise, with two branches that share *S_0_* as the lower reference and use the appropriate saturation area (*S_max+_* or *S_max−_*) as the upper reference:

(2) $$v_{H}=\left\{\begin{array}{l} \frac{S_{H}-S_{0}}{S_{\max^{+}}-S_{0}}(-H_{c}<H\leq H_{t})\\ \frac{S_{H}-S_{0}}{S_{\max^{-}}-S_{0}}(-H_{t}\leq H<-H_{c}) \end{array}\right.\;,$$

where *S_H_* is the aligned-domain area at field *H*; ±*H_t_* is the threshold field at which domain-wall motion completes; *S_max+_* and *S_max−_* are the aligned-domain areas at the positive and negative threshold fields, respectively; and *S_0_* is the aligned-domain area at *H* = −*H_c_*, where the macroscopic magnetization vanishes, the aligned and reverse domains occupy equal areas, and the demagnetizing field reaches its minimum. The point *H* = −*H_c_* is taken as the reference rather than *H* = 0 because the latter corresponds to a remanent state with finite magnetization, which does not represent a true demagnetized configuration of the magnetization process.

By construction, Equation (2) yields υ_H_ = 0 at *H* = −*H*_c_. This is the lower normalization endpoint of the coefficient rather than a true vanishing of the local demagnetizing field, since the multi-domain configuration at *H* = −*H*_c_ still sustains a finite *H*_d_. This does not affect the model here: as the unit magnetization vector m also vanishes in the demagnetized state, the m factor in Equation (3) keeps the contribution small regardless of the exact value of υ_H_.

**Figure 7:**
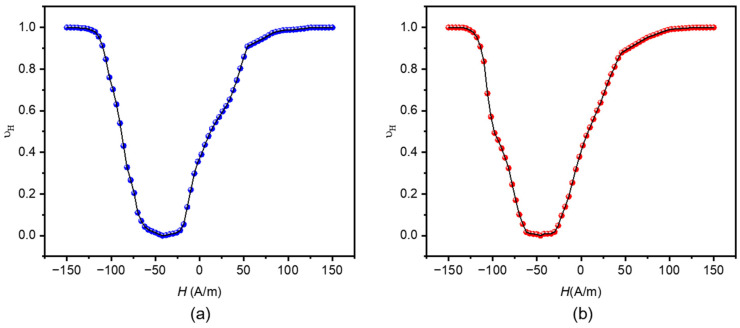
The change in the correction coefficient with the magnetic field (Sample A): (**a**) Zone 1; (**b**) Zone 2.

For Equation (2), two points should be clarified:

(1) The first concerns the linear relation between the aligned-domain area *S_H_* and the demagnetizing field ***H****_d_*, which follows from ***H****_d_* ≈ ***N****_d_*·***M*** during planar domain-wall motion. The approximation holds in the intermediate magnetization range but breaks down at the two endpoints of the υ_H_ curve. Near *H* = ±*H_t_*, domain-wall pinning makes the *S_H_*–***H****_d_* relation nonlinear.

(2) The second concerns the spatial scale of the model. The magnetization direction is resolved at a single representative grain orientation, whereas the S*_H_*(*H*) data are extracted from a Kerr window within an individual grain. Although confined to one grain, the domain-wall motion there is not a purely intra-grain process: the wall responds to an effective field combining the applied field, the demagnetizing field from the surrounding grains, and the local exchange coupling at the grain boundaries. The measured S*_H_*(*H*) curve therefore already embeds the influence of inter-grain magnetostatic interactions on the magnetization progress of the observed grain [7]. The consistent υ_H_ obtained from two differently oriented grains (Figure 7) confirms that the single-grain measurement is representative for the present sample.

Equation (2) thus scales the magnetostatic energy term by the fraction of domain-wall motion completed at each field step, yielding:

(3) $$W_{\mathrm{st}\text{-}\mathrm{revise}}=-v_{H}\cdot \frac{\mu_{0}M_{s}}{2}H_{d}\cdot m\;.$$

## 4. Improved Hysteresis Model and Verification

### 4.1. Domain Energy

The total energy of each domain is the sum of four contributions:

(4) $$W_{total}=W_{zeeman}+W_{an}+W_{st\text{-}revise}+W_{h\;},$$

The Zeeman energy (5) tends to align each domain with the external field; the revised magnetostatic energy (6) corrects the demagnetizing contribution according to the domain area variation derived in Section 3; the magneto-crystalline anisotropy energy (7) constrains magnetic moments to crystal easy axes; and the hysteresis energy (8) is introduced to reproduce hysteresis characteristics following an energy-based approach for ferromagnetic materials [16,17]:

(5) $$W_{zeeman}=-\mu_{0}M_{s}(\alpha_{1}H_{1}+\alpha_{2}H_{2}+\alpha_{3}H_{3})\;,$$

(6) $$W_{st\text{-}revise}=v_{H}\frac{\mu_{0}M_{s}^{2}}{2}(k_{x}m_{x}^{2}+k_{y}m_{y}^{2}+k_{z}m_{z}^{2})\;,$$

(7) $$W_{an}=K_{1}({\alpha_{1}}^{2}{\alpha_{2}}^{2}+{\alpha_{2}}^{2}{\alpha_{3}}^{2}+{\alpha_{3}}^{2}{\alpha_{1}}^{2})+K_{2}({\alpha_{1}}^{2}{\alpha_{2}}^{2}{\alpha_{3}}^{2})\;,$$

(8) $$W_{h}=-\mu_{0}\frac{M_{S}}{\chi_{0}}(\alpha_{1,i}m_{x-p}+\alpha_{2,i}m_{y-p}+\alpha_{3,i}m_{z-p})\;,$$

where k_x_, k_y_, k_z_ are demagnetization factors (i.e., the demagnetizing factors N_x_, N_y_, N_z_ in standard notation), namely the diagonal elements of the diagonalized demagnetizing tensor ***N*** = diag(k_x_, k_y_, k_z_), with the demagnetizing field related to the magnetization by *H*_d,i_ = −k_i_ *M*s *m*_i_ (i = x, y, z). Since the thickness of the electrical steel sheet is much smaller than its in-plane dimensions, it can be treated in the limiting case of an oblate ellipsoid whose normal axis is much shorter than the two in-plane axes: the in-plane demagnetizing coefficients are close to zero, the through-thickness coefficient is the largest, and the three coefficients sum to approximately unity; (*m*_x_, *m*_y_, *m*_z_) is the magnetization vector sum of the domain unit; α_1_, α_2_, α_3_ are the direction cosines of the magnetization vector on the crystal easy axes; K_1_, K_2_ are the magneto-crystalline anisotropy constants; χ_0_ is the initial magnetic susceptibility; and (*m*_x-p_, *m*_y-p_, *m*_z-p_) is the magnetization vector of the domain unit at the previous time step.

### 4.2. Mesoscopic Physical Model

Each crystal grain is composed of six domain types along the easy-axis directions [6]. The six-domain structure here originates from the cubic magnetocrystalline anisotropy of body-centered-cubic Fe-Si: the cubic system possesses three equivalent easy axes along the <100> directions, each associated with two opposite magnetization directions, so that each grain comprises six domain types oriented along the easy axes. In grain-oriented electrical steel, the grains are highly aligned in the Goss texture {110} <001>, with their <001> easy axes strongly aligned to the rolling direction; the single representative grain adopted in the present model is therefore taken in the Goss orientation, consistent with the texture characteristics of grain-oriented electrical steel.

**Figure 8:**
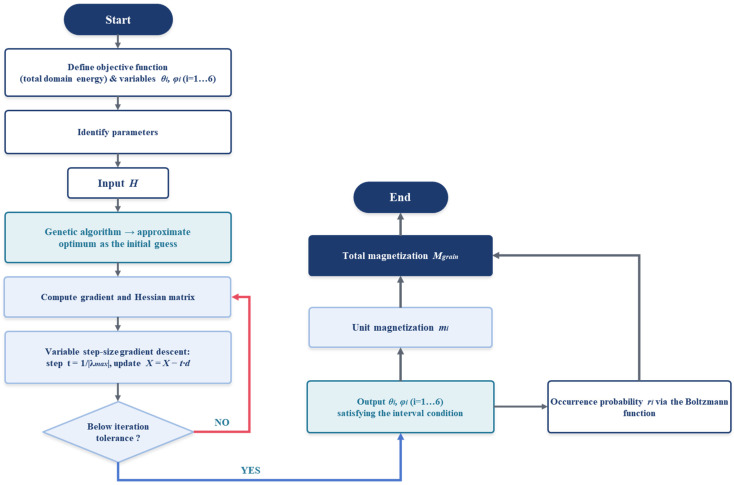
Flowchart of the model solution algorithm.

In the ADSM, an artificial state equation is constructed by summing the total energy of the six domains within a grain and applying gradient descent to find the energy minimum. The local minimum yields the unit magnetization vector mi of each domain and the occurrence probability r_i_ of each domain, from which the magnetization state at each point on the hysteresis loop is obtained. The detailed solution procedure is shown in Figure 8. The unit magnetization vector of the i-th domain is expressed as:

(9) $$m_{i}=[\alpha_{1,i},\alpha_{2,i},\alpha_{3,i}]=\;\left[sin\theta_{i}sin\phi_{i},\;sin\theta_{i}cos\phi_{i},\;cos\theta_{i}\right]\;,$$

where θ_i_ is the angle between the unit magnetization vector and the crystal easy axis [001], and φ_i_ is the angle between the projection of the unit magnetization vector on the (110) plane and the crystal easy axis [010].

The domain occurrence probability r_i_ is expressed via the Boltzmann distribution [9], separated into contributions from hysteresis energy and from all other energy terms:

(10) $$r_{i}=r_{\alpha ,i}r_{h,i}/\sum_{i=1}^{6}(r_{\alpha ,i}r_{h,i})\;,$$

(11) $$r_{h,i}=exp(-\beta A_{s}W_{h,i})/\sum_{i=1}^{6}exp(-\beta A_{s}W_{h,i})\;,$$

(12) $$r_{\alpha ,i}=\frac{exp(-A_{s}(W_{st,i}+W_{an,i}+W_{zeeman,i}))}{\sum_{i=1}^{6}exp(-A_{s}(W_{st,i}+W_{an,i}+W_{zeeman,i}))}\;,$$

where β is the correction coefficient, and A_s_ measures crystal regularity:

(13) $$A_{s}=(3\chi_{0})/(\mu_{0}M_{s}^{2})\;.$$

The total magnetization in the grain is:

(14) $$M_{grain}=\sum_{i=1}^{6}r_{i}M_{s}m_{i}\;.$$

### 4.3. Result Verification and Quantitative Evaluation

#### 4.3.1. Repeatability Validation of the Same Sample

**Figure 9:**
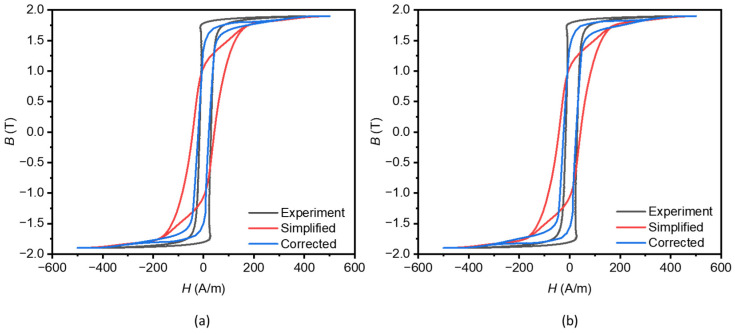
Comparison of the simulation results to the experimental results (Sample A): (**a**) Zone 1; (**b**) Zone 2.

Figure 9 compares the simulated hysteresis loops obtained with (i) the proposed domain-area-corrected magnetostatic energy and (ii) the simplified magnetostatic energy against experimental measurements for both observation zones. The simulation direction follows the field application sequence used in the domain-area experiments: −*H*_max_ ← 0 ← +*H*_max_.

Table 1 compares the coercivity *H*_c_ and remanence *B*_r_ predicted by the simplified model and the corrected model with the experimental values for the two observation regions. The simplified model yields identical values for both regions (*H*_c_ = 49 A/m, *B*_r_ = 1.01 T), deviating from the measured *H*_c_ (23 A/m) and *B*_r_ (1.68 T) by about 113% and 40%, respectively. After applying the correction proposed in Section 3, the predicted *H*_c_ drops to 25 A/m for region 1 and 28 A/m for region 2, corresponding to relative errors of 8.7% and 21.7%; the predicted *B*_r_ rises to 1.39 T and 1.43 T, with errors of 17.3% and 14.9%. The corrected model therefore reproduces the experimental coercivity and remanence with substantially improved accuracy compared with the simplified model.

(1) Coercivity *H*_c_

The simplified model overestimates *H*_c_ by about 113%, giving 49 A/m against the experimental average of 23 A/m. Because the magnetostatic energy is held at its domain-wall-complete maximum throughout the loop, the already-aligned domains experience an unrealistically large demagnetizing pressure at intermediate fields. On the reverse-field sweep this extra resistance requires a much larger reverse field to trigger large-scale domain-wall reversal, thereby pushing *H*_c_ upward. After the correction is applied, υ_H_ < 1 at intermediate states reduces the magnetostatic energy to a level consistent with the actual domain area observed in the Kerr images (Figure 5), and the reversal resistance returns to its proper magnitude. The corrected predictions are 25 A/m for region 1 and 28 A/m for region 2, with residual errors of 8.7% and 21.7% relative to the experimental average.

(2) Remanence *B*_r_

For the remanence *B*_r_, the simplified model underestimates the value by 39.9%, giving 1.01 T against the measured 1.68 T. At *H* = 0, the overestimated magnetostatic energy acts as an effective demagnetizing field that energetically favours additional reverse domains, thereby lowering the net magnetization. After the correction is applied, υ_H_ at *H* = 0 is referenced to S_0_ measured at *H* = − *H*_c_, so that the zero-field magnetostatic energy is no longer pinned to its domain-wall-complete maximum but instead reflects the actual progress of domain-wall motion between the − *H*_c_ reference state and saturation. The predicted zero-field magnetostatic energy therefore comes into agreement with the reverse-domain population observed in the Kerr images. The corrected *B*_r_ is 1.39 T for region 1 and 1.43 T for region 2, with residual errors of 17.3% and 14.9%. The improvement is comparable between the two regions, again confirming the repeatability of the correction.

(3) Residual error and its origins

The residual discrepancy between the corrected simulation and the experiment is consistent with the hybrid-scale nature of the present model discussed in Section 3: υ_H_ carries multi-grain information about how magnetization progresses with field, whereas Equation (9) resolves the magnetization direction at a single representative grain. This single-grain treatment is compatible with the texture of grain-oriented electrical steel, whose grains are highly aligned in the Goss orientation; the Goss texture is nonetheless not perfect, and the slight orientational spread of the grain easy axes about the rolling direction is one possible source of the residual error in *H*_c_ and *B*_r_. Such spread constitutes a minor refinement term rather than a principal deficiency, and the validity of the proposed correction does not depend on resolving it. Introducing an orientation distribution measured by EBSD in place of the single representative grain in Equation (9) may characterize this slight spread and thereby further reduce the discrepancy; its quantitative effect on *H*_c_ and *B*_r_, however, remains to be verified, and its incorporation—reconstructing the single-grain framework into an orientation-distribution framework—constitutes a separate study left for future work.

#### 4.3.2. Cross-Sample Applicability Verification

**Figure 10:**
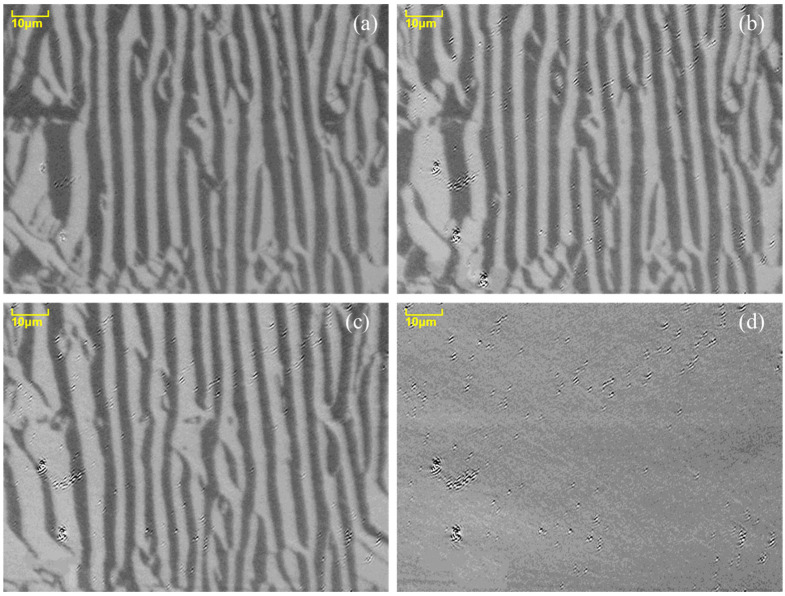
Magnetic domain image (Sample B): (**a**) Initial multi-domain state; (**b**) Domain wall motion; (**c**) Major domain expansion; (**d**) Single-domain saturation.

**Figure 11:**
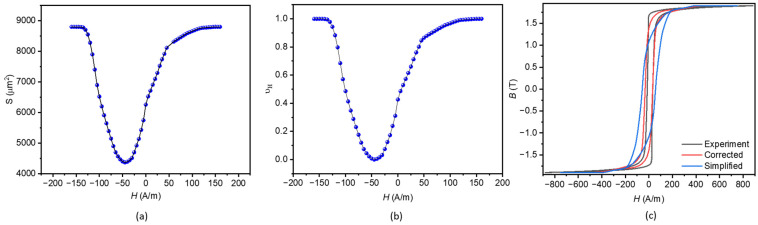
Cross-sample applicability verification: (**a**) Domain area change. (**b**) The change in the correction coefficient with the magnetic field. (**c**) Comparison of the simulation results to the experimental results.

Sample B was observed and processed in exactly the same way as Sample A. Figure 10 shows its domain structure at four representative stages of the magnetization process: the initial multi-domain state, domain-wall motion, major domain expansion, and single-domain saturation. For Sample B, the domain-area curve and the derived correction coefficient υ_H_ are shown in Figure 11a and Figure 11b, respectively, both retaining the same characteristic U-shaped profile as in Sample A. Figure 11c compares the measured hysteresis loop of Sample B with the two model predictions—one using the proposed domain-area-based magnetostatic-energy correction, the other using the simplified magnetostatic energy. The measured loop gives a coercivity *H*_c_ of 26.1 A/m and a remanence *B*_r_ of 1.71 T. The simplified model holds the demagnetizing field at its domain-wall-complete maximum over the whole loop, so it overestimates the magnetostatic energy at intermediate states and returns *H*_c_ = 57 A/m and *B*_r_ = 1.08 T, off the measured values by about 117% and 37%—too high in coercivity and too low in remanence. With the correction applied, the model gives *H*_c_ = 32 A/m and *B*_r_ = 1.39 T, cutting the errors to 22.6% and 18.4%. Both quantities are therefore predicted far more accurately than by the simplified model. The same improvement seen earlier on Sample A thus carries over to Sample B, showing that the correction is not tied to one particular grade.

### 4.4. Comparison with Existing Methods and Discussion

To further clarify the position of the present method, it is compared below with the categories of hysteresis-modeling approaches cited in the Introduction.

Methods for modeling the hysteresis characteristics of electrical steel sheets can be broadly divided into phenomenological models and physical models. Phenomenological models, represented by the Preisach model [4] and the Jiles–Atherton model, are relatively mature and well established in engineering practice and achieve high accuracy in fitting macroscopic hysteresis loops; however, they are essentially mathematical descriptions of the input–output relationship, lack a representation of the underlying magnetization mechanism, and generally rely on the identification and fitting of a large number of parameters. By contrast, the ADSM-type mesoscopic physical models to which the present work belongs start from the variation in domain energy during magnetization, can directly resolve the domain magnetic-moment vectors, and reflect their evolution at different stages of magnetization; they are therefore more advantageous in revealing the magnetization mechanism, at the cost of an accuracy and engineering maturity that, at present, do not yet match those of the well-developed phenomenological models. This trade-off is consistent with the current stage of development of such models.

Within the ADSM framework itself, existing studies have different emphases [6,7]. References focus mainly on simplifying and accelerating the solution of the model—for example, employing a partially implicit method to efficiently solve the artificial state equation. The focus of the present work is different: it addresses the specific limitation that, in the ADSM, the magnetostatic energy has long been treated with a fixed demagnetizing-field approximation, and it introduces a correction coefficient υ_H_ obtained from the measured evolution of the domain area, so that the magnetostatic energy varies dynamically with the domain structure throughout the magnetization process. Compared with studies that start from an energy framework but emphasize magneto-elastic coupling and multi-scale homogenization [8,9], the present work offers a practical route for extracting the field dependence of the magnetostatic energy directly from experimental observation.

Overall, the value of the present method lies not in pursuing the highest fitting accuracy, but in providing a quantifiable, measurement-based dynamic correction for an energy term that has long been treated as fixed in the ADSM, while preserving the clear physical interpretability of a mesoscopic physical model. As noted in the Introduction, an appropriate combination of such mesoscopic physical models with mature phenomenological models to form a multi-scale model is expected to further broaden the applicability of hysteresis models under complex magnetization conditions.

## 5. Conclusions

A simplified, field-independent magnetostatic energy term distorts the simulated hysteresis loop in mesoscopic domain energy models. The root cause is that the demagnetizing field *H*_d_ evolves with the domain configuration during magnetization rather than remaining fixed at its domain-wall-complete maximum.

To address this, the magnetostatic energy is rescaled at each field step by a coefficient υ_H_, defined piecewise with the minimum aligned-domain area S_0_ at *H* = −*H*_c_ as the lower reference and the saturation areas S_max±_ at the threshold fields ±*H*_t_ as the upper references. The coefficient is obtained directly from magneto-optical Kerr microscopy and, through the linear relation ***H***_d_ ≈ ***N***_d_·***M*** valid in the intermediate magnetization range, approximates the fractional growth of the demagnetizing field during domain-wall motion. Although υ_H_ is extracted at the single-grain scale, the domain-wall motion within the observation window responds to an effective field that already contains contributions from the surrounding grains; υ_H_ therefore implicitly reflects the influence of inter-grain magnetostatic interactions on the field-dependence of magnetization progress, without requiring an explicit grain-by-grain calculation. The single representative grain adopted here is compatible with the highly aligned Goss texture of grain-oriented electrical steel, so that the orientation distribution of the texture is not resolved by the model. As the Goss texture is not perfect, the slight orientational spread of the grain easy axes is a minor refinement term rather than a principal limitation; resolving it explicitly—for example by incorporating an EBSD-measured orientation distribution—may further improve the accuracy but remains to be verified and is left for future work.

Compared with experiments on the same sample, the corrected model reproduces both the coercivity and the remanence with substantially improved accuracy. For Sample A, the simplified model gives identical predictions for the two regions (*H*_c_ = 49 A/m, *B*_r_ = 1.01 T), deviating from the measured *H*_c_ (23 A/m) and *B*_r_ (1.68 T) by about 113% and 40%, respectively. After applying the proposed correction, the predicted *H*_c_ drops to 25 A/m for region 1 and 28 A/m for region 2, with relative errors of 8.7% and 21.7%; the predicted *B*_r_ rises to 1.39 T and 1.43 T, with errors of 17.3% and 14.9%. The consistency of these results between the two regions of Sample A, taken from different grains, indicates that the correction is reproducible across different grains. The method was further verified on Sample B (a different, conventional grade), where the errors in *H*_c_ and *B*_r_ were likewise reduced (to about 22.6% and 18.4%, respectively) relative to the simplified model, showing that the correction is applicable across different grades. The small differences between the two regions of Sample A are consistent with local variations between grains rather than with differences in crystallographic orientation.

## Figures and Tables

**Table 1:** Quantitative comparison of coercivity (*H*_c_) and remanence (*B*_r_).

Parameter	Zone	Simplified	Corrected	Experiment	Error (S)	Error (C)
*H*_c_ (A/m)	1	49	25	23	113%	8.7%
*H*_c_ (A/m)	2	49	28	23	113%	21.7%
*B*_r_ (T)	1	1.01	1.39	1.68	39.9%	17.3%
*B*_r_ (T)	2	1.01	1.43	1.68	39.9%	14.9%

## Data Availability

The original contributions presented in this study are included in the article. Further inquiries can be directed to the corresponding author.
